# The Influence of the Menstrual Cycle on Electrical Thresholds for Sensory and Pain Perception: Implications for Exercise and Rehabilitation in Women With and Without Primary Dysmenorrhea—A Pilot Study

**DOI:** 10.3390/healthcare13111240

**Published:** 2025-05-24

**Authors:** Ana Cristina Morales-Lalaguna, Izarbe Ríos-Asín, Pilar Pardos-Aguilella, Jorge Pérez-Rey, Elena Estébanez-de-Miguel, Miguel Malo-Urriés

**Affiliations:** PhysiUZerapy Health Sciences Research Group, Health Sciences Faculty, Department of Physiatry and Nursing, University of Zaragoza, 50009 Zaragoza, Spain; anamoraleslalaguna@gmail.com (A.C.M.-L.); malom@unizar.es (M.M.-U.)

**Keywords:** menstrual cycle, primary dysmenorrhea, electrical thresholds, pain perception, exercise, rehabilitation

## Abstract

**Background**: Hormonal fluctuations during the menstrual cycle (MC) influence pain perception, potentially affecting exercise performance and rehabilitation in women. This effect may be more pronounced in individuals with primary dysmenorrhea (PD), requiring tailored physiotherapeutic and exercise interventions. **Objective**: To analyze the influence of MC phases on sensory electrical threshold (SET) and pain electrical threshold (PET) in eumenorrheic women with and without PD, considering the potential implications for physical activity and rehabilitation. **Methods**: An observational longitudinal study was conducted with 34 physically active women, divided into a control group (CG) and a PD group. SET and PET were measured using transcutaneous electrical nerve stimulation (TENS) at the forearm (peripheral site) and lower abdomen (pain-referred site) across five MC phases. Pain intensity was assessed using a Visual Analog Scale (VAS). **Results**: SET and PET were significantly lower in the premenstrual phase (*p* < 0.001), suggesting increased pain sensitivity. VAS scores were higher in the PD group during all phases, except for the follicular phase (*p* < 0.033), with the highest pain levels recorded in the menstrual and premenstrual phases. While no significant differences in SET and PET were found between groups across most phases, the PD group exhibited a significantly higher SET in the forearm during the premenstrual phase (*p* = 0.005), potentially indicating altered central pain modulation. **Conclusions**: MC-related hormonal fluctuations affect pain sensitivity, particularly in women with PD. These findings underscore the need for phase-specific exercise adaptations and rehabilitation strategies to improve performance, pain management, and recovery in physically active women.

## 1. Introduction

The menstrual cycle (MC), defined as the period from the onset of one menstrual period to the start of the next [1,2], is divided into several phases during which physiological, physical, and behavioral changes occur. Gonadal hormones play a crucial role in these changes [3], as they influence the nervous system and have receptors distributed across various brain regions, including those involved in pain perception and transmission. Thus, hormonal fluctuations—particularly in estrogen and progesterone—may modulate nociception, mood, and peripheral sensitization, thereby influencing pain experience and sensitivity [4,5]: an increase in hormone levels potentiates the endogenous opioid system, acting as an analgesic, while a decrease may increase pain sensitivity [6]. Additionally, female hormones modulate vasodilation and inflammatory processes, directly affecting function and recovery [7]. Thus, the physiological and sensory changes across the menstrual phases (MPs) influence both performance and recovery outcomes in female athletes and active women [8,9].

Hormonal variations and performance-related implications may be even more pronounced in conditions such as dysmenorrhea [10]. Primary dysmenorrhea (PD), defined as cramp-like pain during menstruation with no identifiable underlying cause, is the most common gynecological symptom in women of reproductive age [11,12]; the condition affects 45–95% of women [13] and is more prevalent in adolescents and young women. PD adversely impacts quality of life, disrupts engagement in daily activities, and is a major cause of disability [11]. Women with PD may experience greater discomfort and somatosensory fluctuations during certain MPs, potentially affecting their ability to engage in regular physical activity or structured rehabilitation programs [14].

Somatosensory changes associated with the MC are commonly evaluated using Quantitative Sensory Testing (QST). However, the findings across current studies remain inconsistent. Some of them reported that women tend to have decreased mechanical thresholds during the premenstrual and luteal phases compared to other phases [15,16], while others found no differences in thresholds throughout the MC [17]. In addition to mechanical thresholds, electrical thresholds are a type of QST that offers a potentially reliable, rapid, and straightforward means of evaluating somatosensory function. There is contradictory evidence regarding the effect of sensory electrical perception threshold (SET) and pain electrical threshold (PET) across different MC phases [18,19]. It is uncertain whether current responses act within the MC. Addressing this issue may provide valuable insights into MC-related sensory changes, thereby guiding more tailored and phase-specific approaches in performance and rehabilitation strategies. Moreover, MC-related sensory changes are known to be pronounced in somatosensory syndromes, such as PD. There is a lack of existing studies regarding how PD influences SET and PET across the MC. This represents a significant clinical gap, particularly considering the growing emphasis on personalized approaches to exercise and wellbeing. A clearer understanding of how sensory and pain perception varies across the MC, especially in women with PD, could support more individualized, effective, and safer interventions. This would allow healthcare professionals to optimize exercise recommendations, adjust training loads, and implement pain management strategies to enhance performance and overall wellbeing in female patients and athletes.

This study aims to describe and compare the influence of MC phases on SET and PET in eumenorrheic, physically active women, analyze changes in SET and PET throughout the MC, and evaluate differences between asymptomatic women and those with PD in response to sensory and pain stimuli. In addition to the primary aim, the secondary objective was to discuss the potential implications that these differences may have for exercise and rehabilitation strategies. To our knowledge, this is the first study to compare SET and PET, both in peripheral and pain-referred areas, between the CG and PD group across different phases of the MC, linking it with potential impacts in physical performance. By identifying specific patterns of sensory perception, this pilot study lays the groundwork for future research in this field.

## 2. Materials and Methods

### 2.1. Study Design

A prospective observational longitudinal study was conducted. The study was approved by the Research Ethics Committee of the Autonomous Community of Aragón (CEICA) (C.I.PI23/646) and received authorization for the processing of personal study data from the Data Protection Unit of the University of Zaragoza (CUSTOS), with reference number RAT 2023-306.

### 2.2. Participants

The study sample consisted of women of reproductive age with a regular MC, including both asymptomatic women and those diagnosed with PD. Participants were recruited through advertisements in a health sciences faculty. Individuals interested in participating contacted the main researcher, who confirmed their eligibility and obtained written informed consent before recruitment.

To be eligible for the study, participants had to be over 18 years and self-report to have a regular MC lasting between 21 and 35 days, along with self-declared good health and no underlying medical conditions that could potentially influence the study outcomes [20]. Additionally, they were required to have a consistently reported regular MC (±7 days) for at least the past six months and be physically active according to World Health Organization, engaging in regular moderate-intensity exercise or sports activities for at least 150 min per week [21]. Only women who had tracked their MC for the previous six months with an app or a diary and provided written informed consent were included in the study.

Participants were excluded if they had a history of chronic disorders, including endocrine, neurological, psychiatric, urogenital, or musculoskeletal conditions. Additionally, women who were currently taking systemic medication or using hormonal contraceptives (such as birth control pills or hormonal intrauterine devices) were not eligible to participate. Finally, the possibility of pregnancy was also considered an exclusion criterion due to the absence of an MC and the potential physiological alterations associated with pregnancy [20].

### 2.3. Procedure

#### 2.3.1. Participant Assessment and Group Allocation

At baseline, participants completed a questionnaire assessing demographic data, MC characteristics, and associated symptomatology. Menstrual symptoms were evaluated using the Menstrual Symptom Questionnaire (MSQ), a validated 25-item tool for assessing menstrual-related complaints that is widely used for dysmenorrhea severity assessment [22]. Pain severity was assessed with the Short-Form McGill Pain Questionnaire (SF-MPQ), a 15-item scale that measures sensory and affective pain dimensions [23] and that has been previously used to assess dysmenorrhea [24]. Based on the responses to these questionnaires at baseline, participants were categorized into two groups using the midpoint of the possible score as the cutoff point [22]: (1) PD group: Participants scoring ≥77 points on the MSQ or ≥25 points on the SF-MPQ; (2) Control group (CG): Asymptomatic women with no significant menstrual symptoms and scoring <77 points on the MSQ and <25 points on the SF-MPQ.

#### 2.3.2. Data Collection

Participants were assessed weekly over one complete MC to obtain threshold measurements across MPs. The following standard 28-day MPs were defined [1]: (i) Menstrual Phase (F1): Days 1–5; (ii) Follicular Phase (F2): Days 6–11; (iii) Ovulatory Phase (F3): Days 12–16; (iv) Luteal Phase (F4): Days 17–23; (v) Premenstrual Phase (F5): Days 24–28.

The procedure was replicated in the different MPs at the same time of the day to minimize potential influences of circadian rhythms. The same researcher was responsible for applying and removing all electrodes to minimize inter-examiner bias and ensure consistency throughout the procedure. Participants were assessed once a week over the course of one month, resulting in four assessments per participant. Phase identification and adjustment based on individual cycle length were performed following data collection.

**Figure 1:**
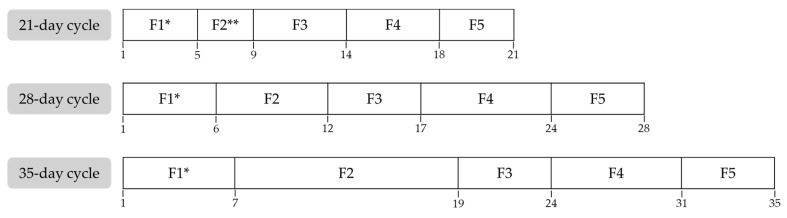
Phase adjustment depending on menstrual cycle duration. * F1 was adjusted based on self-tracking data. ** F2 was modified to a minimum duration of three days; if the total menstrual cycle was shorter, the durations of the remaining phases were proportionally reduced. F1: Menstrual Phase; F2: Follicular Phase; F3: Ovulatory Phase; F4: Luteal Phase; F5: Premenstrual Phase.

Phase identification was determined based on the self-reported first day of menstruation and prospective self-tracking during the following month. In participants with cycles longer or shorter than 28 days, an individualized phase adjustment was performed by adding or subtracting extra days to the F2 (Figure 1), as this is the most variable phase of the MC [2]. No hormonal markers were used to identify MPs. However, as participants reported regular MCs over the previous six months, no alterations in the physiological patterns of normal hormonal fluctuations were expected. 

#### 2.3.3. Study Variables

The primary variables evaluated in this study were the SET and PET, which were measured using transcutaneous electrical nerve stimulation (TENS) with two disposable, square electrodes of 25 cm^2^ each. Throughout the procedure, participants remained in a standardized supine position, with their dominant forearm in supination.

Measurements were taken at two anatomical sites. The first measurement was recorded on the dominant forearm as a representative site for sensory perception in a non-affected peripheral area, which is widely used in the literature as a standard site to measure somatosensory functions. Electrodes were placed on the anterior surface, aligned along the longitudinal axis of the wrist flexor muscle. The distal electrode was positioned 4 cm proximal to the wrist joint line, and the proximal electrode was placed 4 cm from the elbow fold [25]. The second measurement was obtained from the lower abdomen, which was identified as the affected region for PD and is commonly referred to as the site of pain. Two electrodes were applied bilaterally at the midpoint between the umbilicus and the anterior superior iliac spine to evaluate pain perception in a referred pain area [26].

A biphasic symmetrical alternating current was administered using a GYMNA MYO 200 electrotherapy device (Bilzen, Belgium), with the stimulation parameters set at a frequency of 100 Hz, a pulse width of 100 μs, and an increment rate of 1 mA per second. The stimulation intensity was gradually increased from 0 mA until the participant reported the SET, defined as the first consciously perceived tingling sensation [27]. The intensity was then further escalated until the PET was reached, identified as the first sensation of pain [28]. Participants verbally indicated the detection of both thresholds, and a prior trial was conducted to ensure they were accustomed to the sensations. Each measurement was performed twice, and the mean value was used for analysis [19]. If the difference between the two measurements exceeded 2 mA, a third trial was performed, and the two closest values were selected for final analysis [29].

Participants were instructed to adhere to their normal lifestyle, including the continuation of their regular physical activity routine, and to refrain from consuming caffeine and alcohol for a period of 24 h prior to the study session. This protocol was implemented to minimize external factors that could influence sensory perception.

Additionally, menstrual symptoms were evaluated in each MC phase using a Visual Analog Scale (VAS) [30].

### 2.4. Statistical Analysis

All statistical analyses were conducted using SPSS Statistics 29.0 (IBM Corp., Armonk, NY, USA). Descriptive statistics were used to summarize demographic and clinical variables, reporting means and standard deviations (SD) for continuous variables. The normality of all continuous variables was assessed using the Kolmogorov–Smirnov test.

Comparisons between the PD and CG groups were performed. Variables that met the assumption of normality were analyzed using parametric tests (independent *t*-test and repeated-measures ANOVA), while variables that did not meet this assumption were analyzed using non-parametric alternatives, specifically the Mann–Whitney U test for between-group comparisons. A two-way mixed ANOVA was conducted to analyze the interaction between MC phase and group (PD vs. CG) regarding SET, PET, and VAS scores. Statistical significance was set at *p* < 0.05.

## 3. Results

### 3.1. Sample Description

**Figure 2:**
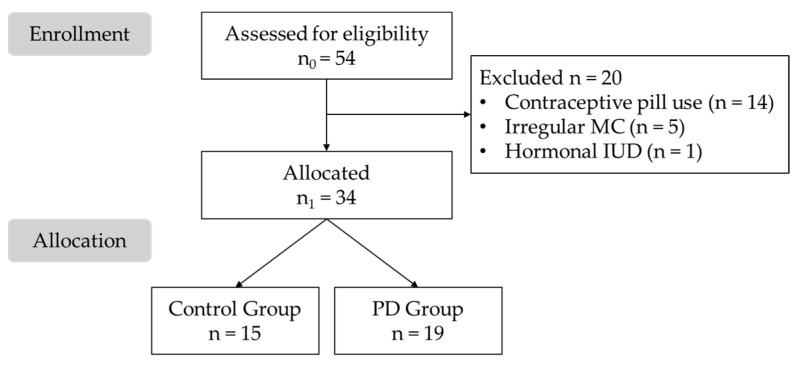
Flow diagram of the study sample. IUD: Intrauterine Device; MC: Menstrual Cycle; PD: Primary Dysmenorrhea.

A total of 54 women were initially recruited for the study. However, 20 participants were excluded due to not meeting the inclusion criteria, leaving a final sample of 34 female health sciences students. Participants were divided into two groups: the CG consisted of 15 asymptomatic women (44.1%), while the PD group included 19 participants (55.9%). A participant flow diagram is provided in Figure 2.

The demographic and clinical characteristics of the sample are presented in Table 1. No statistically significant differences were found between the groups in terms of age, height, or weight, ensuring comparability for further analysis (*p* > 0.05 for all variables).

Regarding menstrual symptomatology, no significant differences were found in the analysis between groups. Nevertheless, the results tend to indicate greater menstrual symptom severity in the PD group. The CG reported an average of 3.73 (SD = 2.87) painful cycles per year, whereas the PD group reported 9.37 (SD = 1.77). Additionally, the MSQ score in the CG was 52.13 (SD = 12.57), compared to 77.21 (SD = 8.75) in the PD group. Similarly, the SF-MPQ score was 11.60 (SD = 9.10) in the CG, while the PD group had a higher mean score of 29.68 (SD = 9.06), reflecting greater pain perception in women with PD.

### 3.2. Descriptive and Comparative Analysis Across Menstrual Cycle Phases

**Figure 3:**
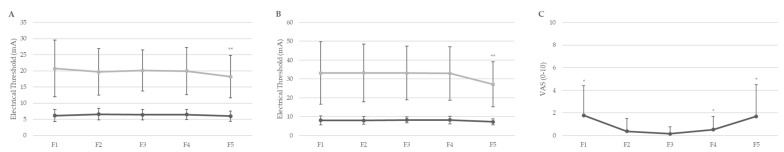
Comparative analysis of sensory and pain thresholds across menstrual cycle phases in entire sample. (**A**) SET (lower) and PET (higher) in non-referred pain area; (**B**) SET (lower) and PET (higher) in lower abdomen; (**C**) VAS scores. F1–F5 refer to the different phases of the menstrual cycle. Data are presented as mean ± standard deviation. * *p* < 0.05; ** *p* < 0.01. PET: Pain Electrical Threshold; SET: Sensory Electrical Threshold; VAS: Visual Analog Scale.

Table 2 presents the variations in SET and PET across the different MPs for the entire sample. A statistically significant reduction (*p* < 0.001) in all SET and PET values was observed during F5, both at the forearm (peripheral site) and the lower abdomen (referred pain site). This decrease in thresholds indicates that a lower current is required to stimulate the nerve fibers, suggesting increased sensitivity to electrical stimulation during this phase of the MC. The results are presented graphically in Figure 3.

Regarding pain perception assessed with the VAS, statistically significant differences were found in F1, F4, and F5 (*p* = 0.002–0.018). The highest pain scores were recorded during phases F1 and F5, which correspond to the phases with the lowest SET and PET values—particularly in F5, where all SET and PET outcomes were significantly lower. This phase-dependent increase in pain sensitivity may have important implications for optimizing physical performance and guiding the development of individualized training and rehabilitation protocols across the MC.

### 3.3. Comparative Analysis Between the Primary Dysmenorrhea and Control Groups

Table 3 presents the SET and PET values at the forearm (peripheral site) and abdomen (referred pain site) across the different MC phases, comparing women with PD and the CG. Additionally, pain perception scores recorded on the VAS (0–10) at each phase are included. Group differences are presented with 95% confidence intervals (CIs) to provide a more precise estimate of effect size and help interpret the presence or absence of meaningful differences between women with and without PD.

No statistically significant differences were found in SET or PET values for either the forearm or abdomen across any phase (*p* = 0.121–0.987), except for SET in the forearm during F5, where the PD group (6.17 ± 2.04) exhibited a higher threshold compared to the CG (5.59 ± 0.97) (*p* = 0.005). No patterns or trends were identified in SET or PET within groups. A statistically significant difference (*p* < 0.001–0.033) in VAS pain scores was observed across all MC phases, with higher pain perception in the PD group in all phases except for F2. Overall, the mean VAS score across all MC phases was 0.32 ± 1.11 (range 0.00–6.00) in the CG and 1.32 ± 2.30 (range 0.00–9.00) in the PD group. The mean VAS scores for each group and phase are detailed in Table 3.

## 4. Discussion

This study aimed to analyze the variations in SET and PET across different MPs in asymptomatic women and those with PD, as well as to examine the influence of these variations on pain perception. The results demonstrated that SET and PET values were significantly lower during F5 in the entire sample, indicating increased sensitivity to electrical stimuli, which was further supported by significantly higher pain perception on the VAS during F1 and F5. Additionally, there were no statistical differences in SET and PET between the PD group and CG, except for SET in F5, which was higher in the PD group.

The results for SET and PET suggest increased pain sensitivity and lower electrical thresholds at the end of the MC. In accordance with our findings, Barbosa et al. (2013) reported lower SET and PET values in a non-referred pain area during F5, attributing these changes to hormonal fluctuations in progesterone and estradiol [19]. Similarly, Bartley et al. (2013) described a lower PET in late F4 compared with F2 in healthy women [31]. It is believed that the drop in progesterone and estrogen during F5 exacerbates premenstrual symptoms, leading to increased pain sensitivity. Elevated estradiol levels are associated with higher pain thresholds during the proliferative phase, whereas the reduction in thresholds observed during F5 is also linked to increased estradiol levels, which further impact emotional and behavioral responses [32]. In line with this, Recacha-Ponce et al. (2023) [33] reported increased electrical thresholds at a peripheral site when estrogen and progesterone levels were elevated, as confirmed by biomarkers. They suggested that women experience greater sensitivity and a lower tolerance to pain in F1 [33]. Another recent study similarly reported that the MPs did not affect the SET, although the means tended to be higher in F3 and F4 [34], which is consistent with our findings. Conversely, other recent studies reported no changes in electrical thresholds throughout the MC. Krunic et al. (2021) [35] found no significant main effects of time or group in eumenorrheic women assessed using PET analysis in a non-referred pain area during the same five MPs used in this study. Nevertheless, they did observe lower cold pain thresholds in F1 compared with F2, F3, and F4, which may align with our results [35]. Caputi et al. (2022) assessed the SET and tolerance pain threshold in F2, F3, and F4 in healthy individuals and did not find differences across MPs [36]. Comparisons with these previous studies are complicated, as they did not analyze electrical thresholds in F5, when gonadal hormone levels rapidly decline and higher rates of migraine headache, temporomandibular disorder, and back pain occur [37]. Supporting our results, other studies had similar findings using mechanical thresholds. A recent study by Fortun-Rabadan et al. (2023) reported lower pain pressure thresholds (PPTs) in F1 and F5 compared to F3, both in healthy individuals and women with PD [38].

When comparing women with and without PD, no statistical differences were found in SET or PET between groups, except for SET at a peripheral site in F5. Aligned with these findings, previous studies reported no differences in pain thresholds in the referred pain area between women with PD and CGs [39,40]. Nevertheless, a recent review (2017) supports the hypothesis that women with PD may report increased pain reactivity (lower pain thresholds) both in referred and non-referred pain areas across different MPs, these differences being heightened in F1 [41]. In line with this, Bartley et al. (2015) reported a lower PET (indicating higher sensory pain to electrocutaneous stimuli) in a population with premenstrual dysphoric disorder [42]. Accordingly, Fortun-Rabadan et al. (2023) reported substantial differences between women with PD and a CG, including a lower PPTs and greater central sensitization in PD, with the differences being more pronounced in F1 and F5 [38]. Electrical thresholds across MPs have been studied in other pain populations compared to CGs. For example, no significant differences were found in SET and PET in individuals with temporomandibular disorders when compared to a CG [35]. In contrast, Tepker et al. (2014) studied electrical thresholds throughout the MC in a migraine population and reported differences in SET, which were higher in the pain group [43]. This may align with our interesting finding observed in F5, where SET in the forearm was significantly higher in the PD group compared to the CG. This differs from the findings of Giamberardino et al. (1997), who reported that dysmenorrhea predominantly exacerbated hyperalgesia in the abdominal muscles and subcutaneous tissues rather than in the extremities [44]. These inconsistencies may be attributed to the widespread sensory sensitivity observed in women with PD, where different QST profiles have been reported compared to CGs [45]. Our results suggest that pain sensitization in PD may not be uniformly distributed across the body and that peripheral sites such as the forearm may exhibit different patterns of modulation depending on the MC phase. These inconsistencies within the current literature may result from methodological differences and the substantial heterogeneity in the outcomes of the cited studies, leaving it uncertain whether electrical thresholds consistently vary between groups.

Another key factor that may influence sensory perception during the MC is the role of endogenous opioids and central sensitization mechanisms. The presence of PD may predispose individuals to chronic pain states due to central sensitization, which could explain part of the hypothesis in this study suggesting that women with PD exhibit increased sensitivity to nociceptive stimuli and reduced pain thresholds [46]. This heightened sensitivity and altered pain processing could further impair exercise tolerance and recovery in women with PD, emphasizing the need for individualized, phase-specific training and rehabilitation adaptations, particularly during high-symptom phases such as F1 and F5 [47]. For instance, clinicians and trainers may consider reducing training intensity, modifying neuromuscular loading, or using painless techniques during these phases to prevent injuries and optimize recovery.

High indices of behavioral variations such as stress, fatigue, anxiety, and mood alterations may also influence pain response through several different neural and physiological mechanisms. Regarding our findings, VAS pain scores were consistently higher in the PD group across all MPs, except for F2. This could be explained by the elevated prostaglandin (PG) levels in F4 compared to F2 during ovulatory cycles [48]. It is well-documented that women with PD have higher levels of PG and that the severity of menstrual pain symptoms is directly proportional to the amount of PG released [49]. This could partially explain the fluctuations in pain perception during MPs and its potential impact on physical performance.

Pain perception and sensory modulation influence neuromuscular control, fatigue, and recovery, which are key factors in both recreational and elite sports settings [26]. Additionally, hormonal levels directly affect cardiovascular, respiratory, neuromuscular, neurocognitive, and metabolic parameters, thus conditioning physical performance [33]. Our findings are of particular importance when considering exercise prescription and sports performance in women. Phase-specific planning may allow more effective training, improving both performance outcomes and injury prevention. It is well established that women experience greater muscle discomfort prior to exercise when estrogen and progesterone concentrations are lower, which may influence their predisposition to engage in intense exercise during this phase [50]. The observed increased pain perception and reduced sensory thresholds in certain MPs may negatively affect exercise adherence, performance, and injury risk, especially in athletes or physically active women who train intensively during these MPs [25,51]. In rehabilitation contexts, these findings suggest that sensory sensitivity and pain perception should be considered when scheduling physical therapy or exercise sessions, particularly in women with PD.

The study offers valuable insights into variations in pain sensitivity across the MC and underscores the need for further research on pain physiology and the hormonal influences on sensory perception. The findings of this study have several practical implications. We identified MC phase-dependent changes in pain sensitivity and sensory modulation, which should be considered when designing exercise and rehabilitation programs for women. The increased pain sensitivity observed during F1 and F5 may negatively impact exercise tolerance, neuromuscular performance, and injury risk—particularly in athletes and physically active women. Individualized exercise adaptations are therefore recommended, with adjustments in training intensity and recovery strategies tailored to the MC phase to minimize discomfort and optimize performance. Furthermore, we observed consistently higher pain perception scores in VAS in the PD group across the majority of MPs. This finding underscores the need for more tailored, phase-adapted interventions to manage symptoms in women with PD, who may experience greater fluctuations in exercise performance and pain tolerance throughout their MCs. Symptom management will also be crucial when designing physiotherapy and rehabilitation sessions, as patients may perceive increased pain in response to certain techniques or treatments, potentially reducing their effectiveness. Lastly, these findings may also contribute to societal implications, including increased adherence to physical activity, better symptom management, and enhanced quality of life for women across different age groups and activity levels.

## 5. Strengths and Limitations

This study has several limitations that should be considered when interpreting the findings. As a pilot study, the results should be interpreted with caution, as this represents an initial step that should be replicated and expanded in future research to be repeated and amplified. Accordingly, the small sample size (n = 34) is justified. Moreover, similar studies have employed comparable sample sizes [33]. Additionally, participants were exclusively health sciences students, which may introduce selection bias and limit the applicability of the findings to the general population. Nevertheless, as PD is a condition highly prevalent among adolescents and young women, we chose to explore sensitivity and pain perception within this specific population. Another limitation is the time constraint, as data collection was restricted to a single MC and was conducted by a single, non-blinded investigator. Furthermore, the MPs were estimated rather than determined through hormonal analysis and dysmenorrhea classification relied on self-reported questionnaires rather than objective diagnostic markers. Additionally, due to logistical constraints, it was not possible to conduct one assessment per MP. Instead, four assessments were conducted per participant throughout the month and the corresponding phase for each assessment was identified retrospectively. Previous studies have determined the MP without blood tests [19], with participants being observed for six months before data collection to confirm the regularity of their cycles. Finally, the lack of assessment of potential confounding factors such as physical performance, psychological state or environmental constraints limits the ability to draw direct conclusions about the specific impact of MPs on sensitivity and pain perception. Nonetheless, measures were implemented to minimize potential sources of bias, including conducting all assessments on the same day of the week and at the same time of day, in the same room, and by the same researcher.

Despite these limitations, it is important to highlight the strengths of the study. Firstly, the accuracy of assessment was ensured by the participants’ prior familiarity with their current sensations. Furthermore, all assessments were conducted by the same researcher, who was blinded to the MP of the participants, thereby significantly reducing potential bias. Secondly, both pain-referred and non-pain-referred areas were evaluated, allowing for a comprehensive assessment of possible peripheral changes in sensitivity. Thirdly, we divided the MC into five phases, including a final premenstrual phase—often omitted in other studies—during which hormonal fluctuations are particularly pronounced and menstruation-related symptoms tend to intensify. Another notable strength is the classification of participants based on responses to two separate questionnaires, ensuring that individuals reporting MC-related pain or symptoms were appropriately assigned to the PD group. Furthermore, our exclusion criteria enhanced hormonal consistency by omitting individuals using contraceptives, those who could potentially be pregnant, or those with known endocrine disorders or conditions that may alter hormonal regulation. Lastly, we used an interdisciplinary approach to discuss not only somatosensory variations throughout the MC but also their potential implications for athletic performance and injury rehabilitation. This helps to explain the gap between pain science and rehabilitation, providing a comprehensive understanding of the relationship between these fields.

The results highlight the importance of future research focusing on the development of MC-phase-specific exercise protocols to optimize training adaptations and support injury prevention strategies. We recommend that future studies include larger and more heterogeneous samples to improve the generalizability of findings. Where feasible, the use of hormonal biomarkers is encouraged to accurately determine the MP, instead of using self-reported measures. Additionally, we suggest the incorporation of multidimensional pain assessment tools that evaluate not only somatosensory aspects but also psychological and cognitive factors to ensure a comprehensive evaluation of pain perception. Assessing physical performance and specifying the type of exercise or physiotherapy applied would also contribute to a more comprehensive understanding of the effects of the MC. Finally, we propose the use of longitudinal designs to enhance the long-term implications of the MC on somatosensory function across multiple cycles. 

## 6. Conclusions

The findings of this study suggest that hormonal fluctuations throughout the MC influence sensory and pain perception, as measured by electrical thresholds. Additionally, PD was associated with heightened pain perception and potential alterations in central or peripheral pain modulation mechanisms. These results underscore the importance of accounting for MC-phase-dependent variations in pain sensitivity when designing personalized rehabilitation and exercise interventions for women, particularly those affected by PD, to optimize performance and reduce the risk of pain-related limitations.

Given that this was a pilot study, further research with larger sample sizes, longitudinal follow-ups, and hormonal analyses is necessary to validate these findings and deepen our understanding of the complex interplay between hormonal fluctuations, sensory modulation, and pain perception in women. Future studies should also refine methodological approaches to control for biological and external factors, ensuring a more comprehensive evaluation of pain physiology and its implications for female health, rehabilitation, and sports performance.

## Figures and Tables

**Table 1:** Demographic and clinical characteristics of the study sample.

	Sample (n = 34)	CG (n = 15)	PD (n = 19)	*p* Value
Age (years)	22.12 ± 4.39	22.27 ± 4.35	22.00 ± 4.53	0.793
Height (cm)	164.71 ± 6.21	163.80 ± 6.77	165.42 ± 5.81	0.570
Weight (Kg)	61.07 ± 10.35	59.40 ± 8.36	62.39 ± 11.74	0.102
Painful cycles	6.88 ± 3.64	3.73 ± 2.87	9.37 ± 1.77	0.327
MSQ	66.15 ± 16.39	52.13 ± 12.57	77.21 ± 8.75	0.147
SF-MPQ	21.71 ± 12.76	11.60 ± 9.10	29.68 ± 9.06	0.855

Values are presented as mean ± standard deviation (SD). “Painful cycles” indicates the number of menstrual cycles per year that participants reported as painful. CG: Control Group; MSQ: Menstrual Symptom Questionnaire; PD: Primary Dysmenorrhea Group; SF-MPQ: Short-Form McGill Pain Questionnaire.

**Table 2:** Comparative analysis of sensory and pain thresholds across menstrual cycle phases.

	F1	F2	F3	F4	F5	*p* Value
SETf	6.14 ± 1.88	6.59 ± 1.81	6.42 ± 1.61	6.45 ± 1.57	5.89 ± 1.61	<0.001 **
PETf	20.72 ± 8.77	19.67 ± 7.21	20.10 ± 6.38	19.91 ± 7.35	18.20 ± 6.56	<0.001 **
SETa	8.04 ± 2.46	8.03 ± 2.12	8.26 ± 1.60	8.22 ± 2.01	7.26 ± 1.59	<0.001 **
PETa	33.10 ± 16.56	33.21 ± 15.31	33.10 ± 14.26	32.95 ± 14.15	27.15 ± 11.94	<0.001 **
VAS	1.79 ± 2.63 *	0.38 ± 1.12	0.16 ± 0.62	0.53 ± 1.17 *	1.70 ± 2.80 *	*p* = 0.002–0.212

Data are presented as mean ± standard deviation (SD). F1–F5 refer to the different phases of the menstrual cycle. * *p* < 0.05; ** *p* < 0.01. PETa: Pain Electrical Threshold in Abdomen; PETf: Pain Electrical Threshold in Forearm; SETa: Sensory Electrical Threshold in Abdomen; SETf: Sensory Electrical Threshold in Forearm; VAS: Visual Analog Scale.

**Table 3:** Comparative analysis of sensory and pain thresholds between the primary dysmenorrhea and control groups across menstrual cycle phases.

MP		CG (n = 15)	PD (n = 19)	*p* Value	CI 95%
F1	SETf	6.32 ± 1.79	6.00 ± 2.01	0.950	(−1.28, 1.92)
	PETf	21.23 ± 11.80	20.32 ± 5.88	0.240	(−6.55, 8.37)
	SETa	7.82 ± 2.03	8.21 ± 2.81	0.121	(−2.48, 1.69)
	PETa	33.77 ± 21.34	32.57 ± 12.47	0.218 †	(−12.89, 15.30)
	VAS	0.58 ± 1.73	2.82 ± 2.88	0.005 * †	(−4.14, −0.33)
F2	SETf	6.50 ± 1.46	6.68 ± 2.17	0.526	(−1.58, 1.22)
	PETf	19.83 ± 7.92	19.50 ± 6.65	0.808 †	(−5.26, 5.93)
	SETa	7.90 ± 1.73	8.18 ± 2.53	0.173	(−1.92, 1.36)
	PETa	31.43 ± 17.02	35.11 ± 13.62	0.987	(−15.47, 8.12)
	VAS	0.73 ± 1.49	0.00 ± 0.00	<0.001 ** †	(−0.09, 1.56)
F3	SETf	5.57 ± 1.55	6.87 ± 1.53	0.743	(−2.41, 0.18)
	PETf	18.20 ± 5.13	21.37 ± 6.97	0.440	(−8.50, 2.17)
	SETa	7.40 ± 1.22	8.83 ± 1.59	0.668	(−2.66, −0.20)
	PETa	26.00 ± 11.83	37.83 ± 14.09	0.511	(−23.03, −0.64)
	VAS	0.00 ± 0.00	0.27 ± 0.80	0.033 * †	(−0.71, 0.18)
F4	SETf	6.45 ± 1.62	6.45 ± 1.58	0.879	(−1.23, 1.24)
	PETf	20.00 ± 7.34	19.87 ± 7.56	0.945†	(−5.67, 5.94)
	SETa	8.27 ± 2.09	8.18 ± 2.02	0.323	(−1.50, 1.67)
	PETa	32.82 ± 15.60	33.03 ± 13.69	0.702	(−11.38, 10.97)
	VAS	0.00 ± 0.00	0.84 ± 1.38	<0.001 ** †	(−1.50, −0.17)
F5	SETf	5.59 ± 0.97	6.17 ± 2.04	0.005 *	(−1.97, 0.82)
	PETf	19.45 ± 8.54	17.04 ± 4.10	0.326	(−3.32, 8.14)
	SETa	7.64 ± 1.47	6.92 ± 1.68	0.532	(−0.65, 2.09)
	PETa	30.05 ± 13.67	24.50 ± 9.96	0.913	(−4.76, 15.85)
	VAS	0.09 ± 0.30	3.17 ± 3.26	<0.001 ** †	(−5.15, −1.00)

Data are presented as mean ± standard deviation (SD). Mean differences between groups are reported with 95% confidence intervals. Normality of data was assessed using the Kolmogorov–Smirnov test. Variables that followed a normal distribution (SET, PET) were analyzed with independent *t*-tests. VAS scores, which did not meet normality assumptions in several phases, were compared using Mann–Whitney U tests. † Mann–Whitney U test applied due to violation of normality assumptions. F1–F5 refer to the different phases of the menstrual cycle. * *p* < 0.05; ** *p* < 0.01. CG: Control Group; CI: Confidence Interval; MP: Menstrual Phase; PD: Primary Dysmenorrhea Group; PETa: Pain Electrical Threshold in Abdomen; PETf: Pain Electrical Threshold in Forearm; SETa: Sensory Electrical Threshold in Abdomen; SETf: Sensory Electrical Threshold in Forearm; VAS: Visual Analog Scale.

## Data Availability

The original contributions presented in this study are included in the article. Further inquiries can be directed to the corresponding author.
